# A nomogram model to predict the portal vein thrombosis risk after surgery in patients with pancreatic cancer

**DOI:** 10.3389/fsurg.2023.1293004

**Published:** 2023-12-19

**Authors:** Jing Wang, Hanxuan Wang, Binglin Li, Songping Cui, Shaocheng Lyu, Ren Lang

**Affiliations:** ^1^ Department of Thoracic Surgery, Beijing Institute of Respiratory Medicine and Beijing Chao-Yang Hospital, Capital Medical University, Beijing, China; ^2^ Department of Hepatobiliary and Pancreaticosplenic Surgery, Beijing ChaoYang Hospital, Capital Medical University, Beijing, China

**Keywords:** portal vein thrombosis, pancreatic cancer, surgery, nomogram, risk

## Abstract

**Background:**

Portal vein thrombosis (PVT) is a common postoperative complication in patients with pancreatic cancer (PC), significantly affecting their quality of life and long-term prognosis. Our aim is to establish a new nomogram to predict the risk of PVT after PC surgery.

**Method:**

We collected data from 416 patients who underwent PC surgery at our hospital between January 2011 and June 2022. This includes 87 patients with PVT and 329 patients without PVT. The patients were randomly divided into a training group and a validation group at a ratio of 7:3. We constructed a nomogram model using the outcomes from both univariate and multivariate logistic regression analyses conducted on the training group. The nomogram’s predictive capacity was assessed using calibration curve, receiver operating characteristic (ROC) curve, and decision curve analysis (DCA).

**Results:**

In the study, the prevalence of PVT was 20.9%. Age, albumin, vein reconstruction and preoperative D-dimer were independent related factors. The model achieved a C-index of 0.810 (95% confidence interval: 0.752–0.867), demonstrating excellent discrimination and calibration performance. The area under the ROC curve of the nomogram was 0.829 (95% CI: 0.750–0.909) in the validation group. DCA confirmed that the nomogram model was clinically useful when the incidence of PVT in patients was 5%–60%.

**Conclusion:**

We have established a high-performance nomogram for predicting the risk of PVT in patients undergoing PC surgery. This will assist clinical doctors in identifying individuals at high risk of PVT and taking appropriate preventive measures.

## 1. Introduction

Pancreatic cancer (PC) is often detected at an advanced stage during its initial diagnosis, with around two-thirds of patients presenting with either locally advanced or metastatic disease. In its early stages, PC is challenging to detect, and the key to long-term survival lies in surgery, with a growing trend of combined resection of invaded venous vessels (1, 2). Venous thromboembolism (VTE) represents a prevalent postoperative complication and ranks as the second leading cause of death among cancer patients (3). Compared with other types of malignancies, pancreatic cancer has the highest risk of VTE, approximately 20% (4). Meanwhile, individuals who experienced VTE following surgery faced a risk of death that was eight times higher (5).

Splanchnic vein thrombosis (SVT), encompassing portal, mesenteric, and splenic vein thrombosis, as well as Budd–Chiari syndrome, is a rare occurrence within the spectrum of VTE. Risk factors for thrombosis include patient-related factors, tumor-related factors, and treatment-related factors (6). This mainly includes age, obesity, prolonged bed rest, tumor type, tumor invasion of veins, tumor stage, chemotherapy, surgery, and venous catheterization, and so on (7, 8), and certain laboratory parameters are also associated with an increased risk of thrombosis, such as white blood cell (WBC) and platelet counts (9, 10). For patients undergoing PC surgery, the occurrence of portal vein thrombosis (PVT) may also be significantly related to the resection and reconstruction of the portal vein system (11). There is no doubt that surgery will increase the risk of PVT occurrence due to venous injury and changes in hemodynamics. The occurrence of PVT can affect the patients’ quality of life and long-term survival (12). Consequently, it is essential to assess the PVT risk and promptly take preventive measures.

The Caprini risk assessment model (RAM) has been most widely used in surgical patients for many years (13). Although Caprini RAM has practicality in identifying VTE risk, it shows insufficient performance in assessing PVT risk in patients undergoing PC surgery. Because according to its grouping criteria, all patients were at high or very high risk. We also assessed some other common scales, but the results were not satisfactory (14).The nomogram is an intuitive predictive tool that enables precise individualized assessment by adding independent correlates and potential biomarkers to make the predictive model more targeted. Nomogram has now been applied to the study of various malignancies and has been shown to be a reliable tool for predicting cancer prognosis, cancer-associated thrombosis (CAT) (15–17).

Therefore, the purpose of this study is to construct a model for assessing the risk of PVT after PC surgery by nomogram to screen out the population at high risk of PVT and provide some reference for clinical decision-making.

## 2. Materials and methods

### 2.1. Ethics approval and consent to participate

All surgical procedures and therapies were consented by patients and their family members. All procedures in this study involving human participants were performed in accordance with the ethical standards of the institutional research committee and the 1964 Helsinki Declaration and were approved by Ethnic Committee and Committee for Clinical Application of Medical Technology of Beijing ChaoYang Hospital, Capital Medical University (No. 2020-D-301).

### 2.2. Patients

We retrospectively analyzed the clinical data and follow-up data of patients that met inclusion criteria. Inclusion criteria: (1) Patients who received surgery in Department of Hepatobiliary and Pancreaticosplenic Surgery in Beijing Chaoyang Hospital from January 2011 to June 2022; (2) Age between 18 and 80, no limitation on gender; (3) Preoperative imaging and postoperative pathological examination confirmed PC; (4) Patients with tumors suffered no arterial invasion and distal metastasis; (5) Clinical data and follow-up data were integral and available. Exclusion criteria: (1) Patients with other systemic tumors; (2) Patients with hematologic diseases.

### 2.3. Clinical characteristics and follow-up

The following data were recorded through the electronic medical record system: patient-related information included age, sex, body mass index (BMI), comorbidities (hypertension, diabetes, coronary heart disease, etc.); tumor-related information included preoperative imaging examination, tumor type, tumor diameter, venous invasion, lymph node metastasis and distant metastasis; treatment-related information included surgical approach, operation time, bleeding, transfusion, vein reconstruction; routine laboratory tests included WBC, neutrophil percentage, hemoglobin, platelet, albumin (ALB), total bilirubin, tumor markers (CA199, CEA, etc.), D-dimer.

The outcome of this study was the occurrence of PVT events in patients within 6 months after surgery. Follow-up examinations primarily include laboratory tests and contrast-enhanced CT scans of the abdomen. All patients were followed up by telephone, return visit, or inpatient observation, and the follow-up data were recorded until December 2022 or death.

### 2.4. Derivation and validation of the nomogram model

To ascertain the risk factors associated with new PVT after PC, the researchers initially performed univariate analysis. Subsequently, variables with a significant *P* value of less than 0.2 were incorporated into a multivariate logistic regression analysis to identify independent risk factors. In the end, nomogram models were created using independent risk factors that were identified through multivariate logistic regression. The calibration curves were employed to illustrate the concurrence between the predicted and actual probabilities. The discriminative capacity was assessed by evaluating a receiver operating characteristic curve (ROC) and computing the area under the curve (AUC). Ultimately, decision curve analysis (DCA) was utilized to evaluate the overall advantage and clinical value for patients. This involved measuring the net benefit across different threshold probabilities within the validation group.

### 2.5. Statistical analysis

In the present research, variables with missing values exceeding 30% were excluded, and the missing data were imputed using a predictive average matching algorithm. The measurement data were reported as either the mean and standard deviation or the median and interquartile range, while count data were presented as numerical values. Measurement data were compared using a two-sample unpaired t-test, while count data were analyzed using either a chi-square test or Fisher’s exact test. Statistical significance was determined at a two-sided *p*-value < 0.05. All the data were analyzed using SPSS (version 26.0) and R software (version 4.2.0).

## 3. Results

### 3.1. Baseline and clinical characteristics of patients

A total of 416 consecutive patients were recruited in this study, and the prevalence of PVT in the present study was 20.9% (87/416). Included 244 males and 172 females with a mean age of 62.5 ± 10.8 years. Patients were randomly divided into 291 patients (70%, 61 PVT patients and 230 non-PVT patients) in the training group and 125 patients (30%, 26 PVT patients and 99 non-PVT patients) in the validation group at a ratio of 7:3. We compared the clinical characteristics of the training and validation groups and showed no significant differences. Clinical characteristics of the two groups are shown in the Table 1.

**Table 1:** Clinical characteristics of the training and validation group.

Variable	Total (*n* = 416)	Training group (*n* = 291)	Validation group (*n* = 125)	*P* value
Age, years	62.5 ± 10.8	63.0 ± 10.8	61.4 ± 10.8	0.160
Gender (males)	244	178	66	0.112
BMI, kg/m^2^	24.0 ± 2.6	23.8 ± 3.2	24.3 ± 2.4	0.320
Comorbidities (yes)	220	148	72	0.207
Operation time, hours	10.3 ± 4.0	10.4 ± 4.1	10.2 ± 3.9	0.667
Bleeding, ml	500 (400)	500 (400)	500 (475)	0.262
Blood transfusion, ml	0 (800)	0 (800)	0 (600)	0.259
Vein reconstruction	190	137	53	0.380
Tumor diameter, cm	3.9 ± 1.8	3.9 ± 1.9	3.8 ± 1.7	0.505
Lymph node metastasis	249	176	73	0.691
WBC, ×10^9^/L	6.1 ± 2.2	6.1 ± 2.2	6.1 ± 2.1	0.874
Hemoglobin, g/L	122.4 ± 17.9	122.3 ± 18.1	122.8 ± 17.6	0.804
PLT, ×10^9^/L	214.1 ± 76.6	216.2 ± 81.3	209.3 ± 64.4	0.419
ALB, g/L	37.6 ± 5.3	37.7 ± 5.2	37.4 ± 5.3	0.704
TB, μmol/L	18.2 (107.5)	19.0 (112.4)	16.1 (98.3)	0.614
CA199, U/ml	213.2 (645.0)	215.8 (819.3)	159.9 (579.0)	0.805
CEA, ng/ml	2.4 (3.1)	2.3 (3.1)	2.6 (3.5)	0.952
Pre-D-dimer, mg/ml	0.7 ± 0.2	0.7 ± 0.2	0.7 ± 0.2	0.782

BMI, body mass index; WBC, white blood cell; PLT, platelets; ALB, albumin; TB, total bilirubin; CA199, carbohydrate antigen199; CEA, carcinoembryonic antigen.

### 3.2. The risk factors associated with VTE in the training group

Univariate and multivariate logistic analyses were performed and subsequently 5 PVT related factors (*P* < 0.2) were identified in Table 2. Among them, age (OR: 2.495, 95% CI: 1.209–5.148, *P* = 0.013), vein reconstruction (OR: 4.966, 95% CI: 2.184–11.290, *P* < 0.001), ALB (OR: 0.877, 95% CI: 0.818–0.941, *P* < 0.001) and pre-D-dimer (OR: 5.913, 95% CI: 1.098–31.834, *P* = 0.039) were found to be independently associated with PVT in patients after PC surgery. Other variables were not significantly different.

**Table 2:** Factors associated with the VTE in the training group.

Variable	Univariate analysis		Multivariate analysis	
	OR (95% CI)	*P* value	OR (95% CI)	*P* value
Age	2.995 (1.539–5.827)	0.001	2.495 (1.209–5.148)	0.013
Gender	1.161 (0.646–2.085)	0.618		
BMI	0.787 (0.692–1.175)	0.645		
Comorbidities	1.126 (0.712–2.042)	0.652		
Operation time	1.125 (1.047–1.209)	0.001	1.035 (0.943–1.136)	0.470
Bleeding	1.000 (1.000–1.001)	0.282		
Blood transfusion	1.000 (1.000–1.001)	0.240		
Vein reconstruction	5.036 (2.622–9.670)	< 0.001	4.966 (2.184–11.290)	< 0.001
Tumor diameter	1.038 (0.897–1.201)	0.616		
Lymph node metastasis	1.682 (0.819–3.581)	0.217		
WBC	0.962 (0.839–1.105)	0.587		
Hemoglobin	1.006 (0.990–1.022)	0.467		
PLT	1.001 (0.997–1.004)	0.768		
ALB	0.894 (0.841–0.950)	< 0.001	0.877 (0.818–0.941)	< 0.001
TB	1.001 (0.998–1.004)	0.506		
CA199	1.000 (1.000–1.000)	0.886		
CEA	0.992 (0.963–1.022)	0.585		
Pre-D-dimer	9.306 (2.114–40.967)	0.003	5.913 (1.098–31.834)	0.039

BMI, body mass index; WBC, white blood cell; PLT, platelets; ALB, albumin; TB, total bilirubin; CA199, carbohydrate antigen199; CEA, carcinoembryonic antigen.

### 3.3. Development and validation of nomogram model

**Figure 1:**
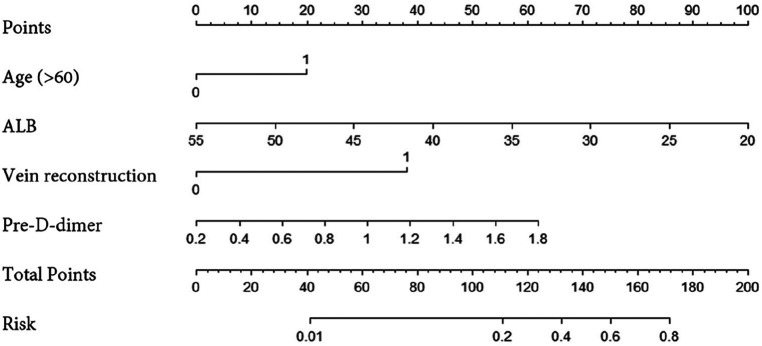
A nomogram model for PVT risk in patients undergoing PC surgery.

We constructed a nomogram using independent factors derived from a multivariate logistic regression analysis of the training group (Figure 1). In this nomogram, individual factors are assigned specific scores, and then the cumulative overall risk score is calculated based on these scores. Afterward, visually estimating the probability of PVT for each patient after PC surgery can be achieved by drawing a straight line downwards.

**Figure 2:**
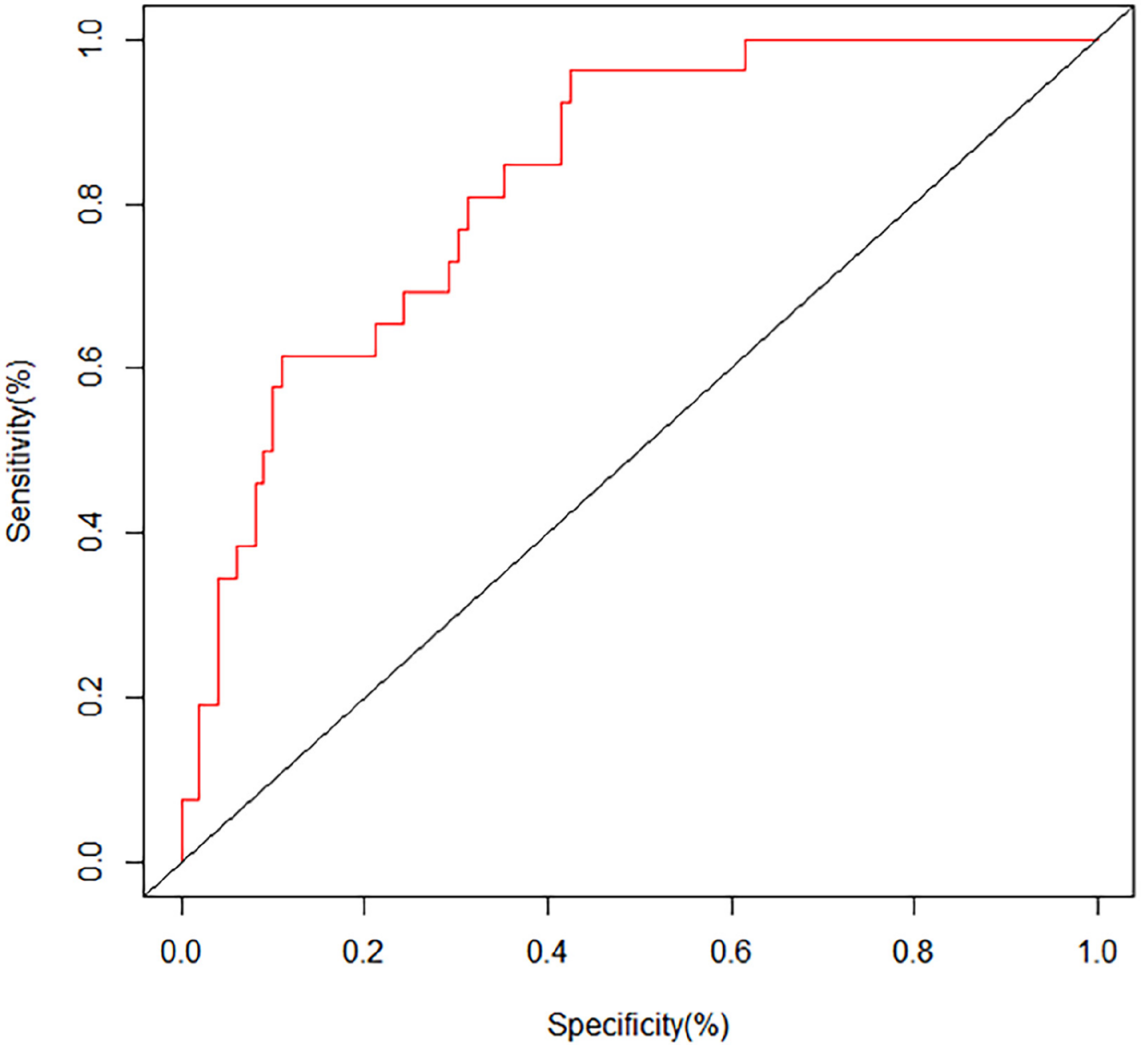
ROC curves of the nomogram of postoperative PVT in patients with PC surgery.

**Figure 3:**
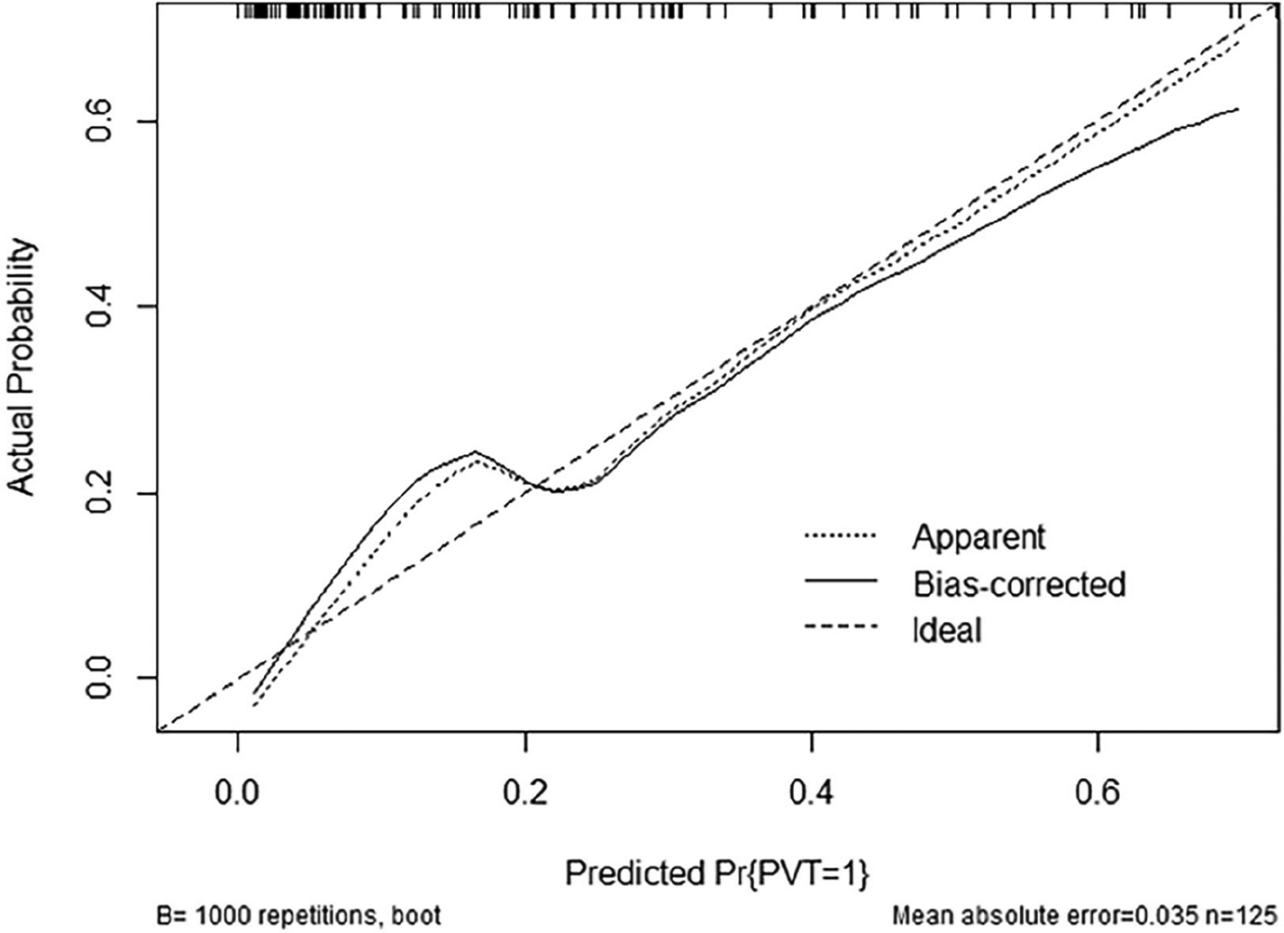
Calibration plot of the nomogram for the probability of PVT in patients with PC surgery (bootstrap 1,000 repetitions).

**Figure 4.**
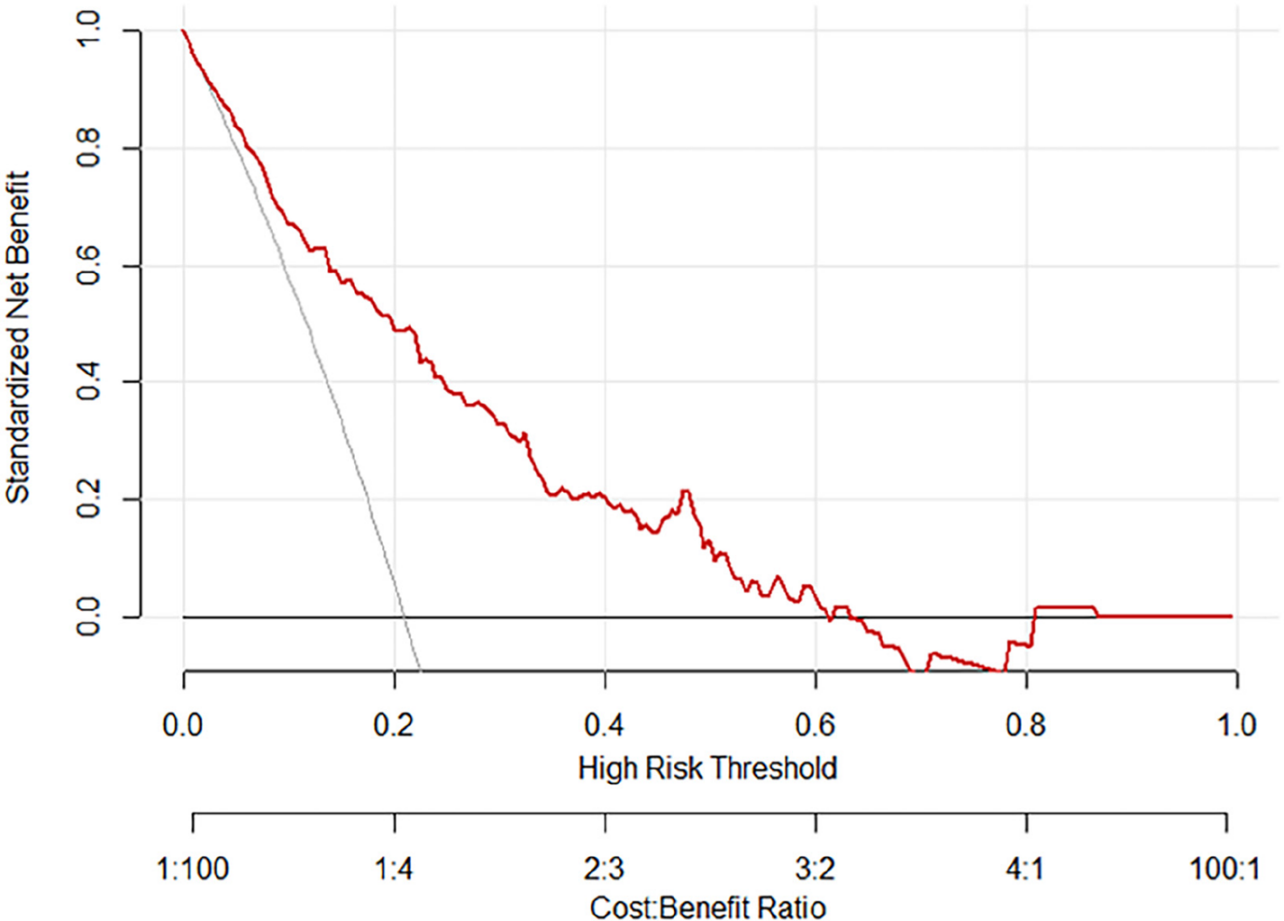
The decision curve analysis for the nomogram of postoperative PVT in patients with PC surgery.

The ROC curves and calibration curves were used to evaluate this nomogram for the validation groups (Figures 2, 3). The nomogram demonstrated a C-index of 0.810 (95% confidence interval: 0.752–0.867). The AUC for the nomogram was 0.829 (95% CI: 0.750–0.909) in validation group (Figure 2). The calibration curve of the nomogram for the prediction of PVT risk in PC patients demonstrated a good agreement in the training group (Figure 3). DCA was conducted to evaluate the clinical usefulness of the prediction nomogram. The findings validated the clinical utility of the nomogram in cases where the occurrence of PVT in patients ranged from 5% to 60% (Figure 4).

## 4. Discussion

At present, surgery is still the only curative treatment for PC. An increasing number of studies have shown that thrombosis is an important cause of postoperative mortality and the second leading cause of death in cancer patients (3, 18, 19). For PC patients, the tumor is more aggressive, the surgery time is longer, and it involves the resection and reconstruction of the portal venous system, which also means a higher risk of VTE, especially PVT. Therefore, we conducted this study to explore the prevalence and related factors of PVT after PC, and to construct a PVT prediction model, which attracted the attention of clinicians and also provided a reference for screening people at high risk of PVT in clinical work. In this study, the prevalence of VTE was observed to be 20.9%. Age, ALB, vein reconstruction and preoperative D-dimer were independent related factors.

Numerous patient-related risk factors are associated with the activation of coagulation and substantially contribute to the risk of thrombosis in cancer patients. These factors include but are not limited to age, gender, race, and common comorbidities (20, 21). In this study, we obtained similar results that age is an independent risk factor for PVT in patients after PC surgery. In addition, this study concluded that patients’ preoperative plasma ALB level was a protective factor. Previous studies have shown that cancer patients with lower serum ALB levels have a significantly increased risk of thrombosis and death compared to cancer patients with higher serum ALB levels (22). The study showed that ALB was less than 44.2 g/L, the risk of thrombosis increased approximately 2-fold. PC patients often suffer from hypoproteinemia due to poor digestive function and tumor consumption resulting in malnutrition. This increases the risk of developing PVT perioperatively. However, the mechanisms involved also need to be explored by further studies.

The risk of developing thrombosis is most influenced by recent surgery. In the Olmstead County series, patients who had recently been hospitalized for surgery had a 22-fold (95% CI, 9.4–49.9) higher risk of thrombosis compared with those who had not recently been hospitalized or had not undergone surgery (23). Surgery-related factors include body position, operation site, operation duration, operation method, blood loss, blood transfusion volume, vascular occlusion time, etc. For patients undergoing PC surgery, portal system occlusion and reconstruction are often performed during surgery, which inevitably causes local arteriovenous injury, while vascular endothelial injury exposes collagen and basement membrane, releases tissue factor (TF), promotes platelet aggregation, and leads to thrombosis (24). Prolonged operation time will lead to prolonged recumbency, but also lead to lower limb muscle pump reflux weakened, lower limb venous dilatation in patients with venous return blocked, blood stasis, prone to thrombosis events. Moreover, coagulation disorders may arise as a consequence of intricate surgical procedures, resulting in significant bleeding and necessitating blood transfusions (25). Surgery has the potential to trigger the body’s inflammatory response, activate neutrophils, and facilitate thrombosis and tumor metastasis (26, 27).

Tumor-related factors encompass aspects such as the type of tumor, its primary location, cancer stage, grade, and more. It is generally believed that the types of cancer most associated with thrombosis are gastric cancer and PC (28, 29). More and more studies have shown that tumor cells directly or grounded to activate the coagulation process by expressing procoagulant factors, cell surface adhesion molecules, as well as activating platelets and inflammatory cells, resulting in a hypercoagulable state of the blood, thereby promoting the occurrence of thrombosis. On the one hand, tumor cells have the ability to release various factors, including TF and intercellular cell adhesion molecule (I-CAM). These factors play a direct role in the coagulation process and actively encourage the formation of CAT (30–33). On the other hand, tumor cells activate normal cells (endothelial cells, platelets, leukocytes, etc.) by synthesizing and releasing soluble regulators or adhesion, so that they undergo corresponding procoagulant changes and promote the formation of thrombosis (34–37).

Within haematologic biomarkers, an increased D-dimer level demonstrates a robust and autonomous correlation with thrombosis in individuals with cancer (38–41). D-dimer results from the breakdown of cross-linked fibrin, serving as a marker for both global coagulation activation and fibrinolysis. Elevated levels of D-dimer mostly reflect a hypercoagulable state induced by hemostasis activation (42). Because D-dimer levels tend to fluctuate greatly due to factors such as surgery, we selected the results of preoperative D-dimer in patients, which can also more acutely reflect the coagulation status of patients. The results showed that preoperative D-dimer level was an independent risk factor for PVT in patients undergoing PC surgery. In addition, other biomarkers independently associated with CAT including plasma soluble P-selectin (sP-selectin) and prothrombin fragment 1 + 2 (F1 + 2), are also worthy of further study.

The Caprini RAM is often used to assess VTE risk in patients after surgical procedures (43). However, all patients undergoing PC surgery were at high risk of Caprini RAM, which also means that Caprini RAM does not distinguish well between this truly high-risk group. Hence, this research aimed to develop a nomogram utilizing independent factors obtained from the analysis. The purpose was to assist clinicians in identifying individuals at a heightened risk of PVT and offer a valuable reference for subsequent preventive measures.

Our study also has some limitations as follows. First, this is a single-center retrospective study, and the research findings require prospective or external validation. Furthermore, the small sample size of the study requires future large-scale multicenter research to further explore and validate these findings. Finally, this study included patients who underwent PC surgery, so whether the results are applicable to other types of patients still needs to be confirmed through further research.

## 5. Conclusion

Age, ALB, vein reconstruction and preoperative D-dimer were independent factors associated with PVT in patients undergoing PC surgery. Our nomogram can assist clinical doctors in screening high-risk individuals with PVT who can benefit from preventive measures like anticoagulation. It also helps avoid complications caused by unnecessary preventive actions.

## Data Availability

The raw data supporting the conclusions of this article will be made available by the authors, without undue reservation.
